# Prevalence of Underweight, Stunting, and Wasting among Children Infected with Human Immunodeficiency Virus in South India

**DOI:** 10.1155/2009/837627

**Published:** 2009-07-02

**Authors:** C. Padmapriyadarsini, N. Pooranagangadevi, K. Chandrasekaran, Sudha Subramanyan, C. Thiruvalluvan, P. K. Bhavani, Soumya Swaminathan

**Affiliations:** Department of Clinical Research, Tuberculosis Research Centre (ICMR), Chennai 600031, India

## Abstract

*Background*. Growth failure is a common feature of children with human immunodeficiency virus (HIV) infection. Malnutrition increases mortality and may impair the response to antiretroviral treatment. *Objective*. Our objective was to describe the prevalence of stunting, underweight, and wasting in HIV-infected children in south India and to assess the utility of these parameters in predicting immune status. *Methodology*. In this cross-sectional study, anthropometric measurements and CD4 counts were performed on 231 HIV-infected children. *Z* scores for height for age, weight for age, and weight for height were correlated with CD4 cell counts and receiver operating characteristic curves plotted. *Results*. Prevalence of underweight was 63%, stunting 58%, and wasting 16%, respectively. 33–45% of children were moderately or severely malnourished even at CD4 >25%; sensitivity and specificity of stunting or underweight to predict HIV disease severity was low. *Conclusions*. Undernutrition and stunting are common among HIV-infected children at all stages of the disease in India. Early and aggressive nutritional intervention is required, if long-term outcomes are to be improved.

## 1. Introduction

The UNAIDS report on the global AIDS epidemic estimated that approximately 420 000 (350 000–540 000) new HIV infections occurred in children below 15 years of age in the year 2007, 90% of them through mother to child transmission [1]. Malnutrition has been shown to be an important comorbid condition, as the same populations that are vulnerable to HIV also have a high prevalence of food insecurity [2]. There is limited data on the prevalence and type of malnutrition (underweight, stunting, and wasting) among HIV-infected children in India [3], though it is known that protein energy malnutrition is one of the commonest manifestations of HIV in this region [4, 5]. While malnutrition itself is multifactorial in causation, the most effective treatment for this failure to grow in HIV-infected children appears to be antiretroviral therapy [6]. Further, the use of growth monitoring in health care settings caring for HIV-infected children could lead to an earlier identification of malnutrition and appropriate interventions, including nutritional counseling, supplementation, and ART [7].

The Tuberculosis Research Centre has been following a cohort of HIV-infected children from 2001 in Chennai and Madurai cities of Tamil Nadu, south India. Data from this study of HIV-infected children provided us with a unique opportunity to describe the prevalence of stunting, underweight, and wasting at presentation and also examine any relationship with age, sex, and stage of the disease. We investigated whether any of these indices of nutritional status could predict the immune status of the child or serve as surrogate markers of disease severity. If so, they could be potentially used in peripheral health care settings, where facilities for sophisticated laboratory monitoring may not be available.

## 2. Materials and Methods

This was a cross-sectional study of children infected with HIV, between the ages of 0 and 15 years, who were referred to the outpatient clinics of Tuberculosis research Centre (TRC) in the cities of Chennai and Madurai, south India between May 2001 and December 2007. Children already on antiretroviral therapy (ART), in a moribund state, not willing for regular hospital visits or blood draw as per the study protocol were excluded. Children were assessed clinically, examined for physical and mental development, nutritional status, and any evidence of opportunistic infections including tuberculosis. Staging was done using the WHO clinical staging chart [8] and CD4 count and CD4 percentage measured by standard flow cytometric methods using the Beckman Coulter Epics XL.

Children were referred for ART initiation if eligible, as per WHO and national guidelines [8]. However, provision of free ART was launched by the government of India in April 2004 and a special pediatric initiative in November 2006. Prior to this, access to ART was limited as very few patients could afford to buy these drugs. The study was approved by the Institutional Ethics committee of TRC and written informed consent was obtained from the parent or legal guardian.

Children were included in this analysis if measurements for height and weight were obtained upon enrollment. The *Z*-scores for weight, height and BMI were computed based on the child's age and gender using the EPI-NUT component of the EPI-INFO 2002 software package (version 3.4.3) from CDC (based on NCHS reference median values). The WHO Global Database on Child Growth and Malnutrition recommends a cutoff *z* score of < −2 to classify low weight-for-age (underweight), low height-for-age (stunting), and low weight-for-height (wasting) as moderate and a *z* score of < −3 standard deviations (SD) to define severe undernutrition [6]. A *z* score of < −2 indicates that a child's height for age (HAZ), weight for age (WAZ), or weight for height (WHZ) is 2 SD below the age and gender-specific median for the normal population.

### 2.1. Data Analysis

SPSS version 11.0 was used for statistical analysis. The prevalence of underweight, stunting, and wasting were calculated for the different age groups and both sexes and the severity classified based on *z* scores. Differences in the proportions of wasting, stunting, and underweight among boys and girls and at various ages were tested with the chi-square test. Correlation between CD4% and growth indices was obtained using Pearson's correlation coefficient. Receiver Operating Characteristic curves were constructed to assess the relationship between HAZ and WAZ with CD4% and to determine the cutoff which would predict immune deficiency with optimal sensitivity and specificity.

## 3. Results

A total of two hundred and thirty one antiretroviral-naïve HIV-infected children were enrolled during the period under study. The average age of the children at presentation was approximately 71 months with 17% under 3 years of age. 42% were boys and majority of the children were in WHO clinical stage 3. The mean CD4 percentage was 17.7 ± 10 (SD)% and the average BMI was 14.2 ± 2 (Table 1).

**Figure 1:**
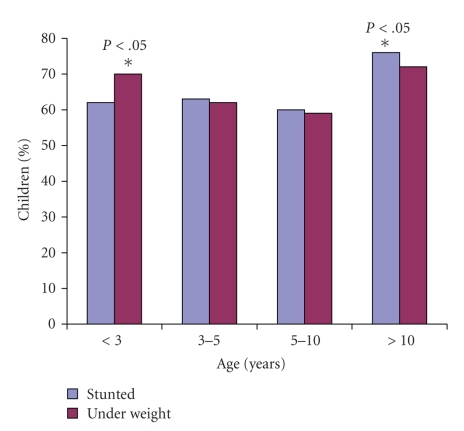
Proportion of children with underweight (WAZ < −2 SD) and stunting (HAZ < −2 SD) in different age groups. *P < .05 versus 3–5 and 5–10 years age group.

In this cohort, the prevalence of underweight (WAZ < −2) was 63%, stunting (HAZ < −2) 58%, and wasting (WHZ < −2) 16%, respectively, (Table 2). While a higher proportion of boys were underweight compared to girls, the rates of stunting and wasting were similar. Figure 1 shows the proportion of underweight and stunted children in the various age categories. Children below 3 years of age and above 10 years of age were at a significantly higher risk of severe underweight when compared with children between the ages 3–10 years but the proportion of stunted children was similar across all age groups. Severe stunting was found in >40% of children at all age groups. The proportion of children with normal nutritional status (WAZ and HAZ > −1) tended to decrease with increasing age. Table 3 shows the mean CD4% and CD4 cell count of all children as well as in the subsets with either underweight or stunting, with a clear trend towards lower cell count and percentage among children above the age of 10 years in all three groups.

**Figure 2:**
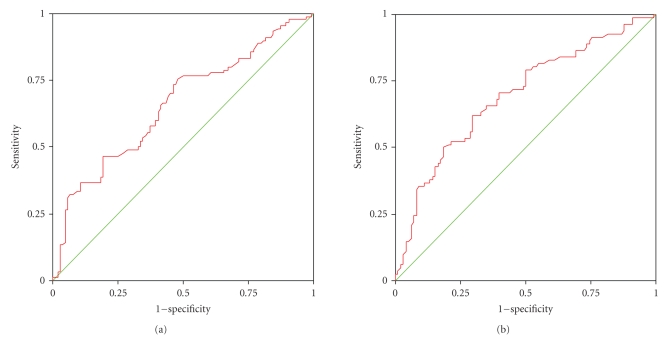
(a) Receiver Operator Characteristic curve between WAZ score and CD4 percentage, and (b) HAZ score and CD4 percentage.

We examined the relationship between CD4 percentage and the various growth parameters. CD4% was available for 194 children with 41% of them showing severe immunodeficiency (CD4 <15%) at the time of initial presentation. Of these children with CD4 <15%, 71% were stunted (HAZ < −2) and 76% were underweight (WAZ < −2). Table 4 shows the prevalence of different types of malnutrition in children at different levels of immunodeficiency. Higher rates of moderate to severe stunting and underweight were observed among children with CD4 <15% (*P* < .001) compared to those at higher CD4 counts. There was a moderate correlation between WAZ and CD4% (*r* = 0.3, *P* < .005) and between HAZ and CD4% (*r* = 0.28, *P* < .005). Even at CD4 counts >25% indicating normal immune status, 33 to 45% of children had moderate to severe malnutrition. The sensitivity and specificity of stunting (HAZ < −2) to predict CD4 <15% was 63% and 67% while undernutrition (WAZ < −2) could predict a CD4 <15% with a sensitivity of 60% and specificity of 61%, respectively. Further, the area under the ROC Curve for WAZ and CD4% was 0.66 (95% CI 0.58–0.74) while for HAZ and CD4% area under the curve was 0.69 (95% CI 0.62–0.77), Figures 2(a) and 2(b).

## 4. Discussion

The overall prevalence of moderate to severe underweight and stunting in this population of HIV-infected children from South India was 63% and 58%, which is cause for concern. In children under 5 years, the prevalence was 66% and 62%, respectively—this is much higher than the national average of 48% underweight and 40% stunting reported by NFHS-3 for under-five children [9]. Our findings are similar to rates of undernutrition among HIV-infected children reported from other parts of India, which vary from 60 to 62% [4, 10]. These figures are higher than those reported among HIV infected children in Africa, which varies between 14% for undernutrition and 31% for stunting to 38% for malnutrition, [11–13]. Our data highlights the much higher rate of moderate and severe grades of malnutrition among HIV-infected children in India. The children included in this report were seeking care at government health facilities and represent the majority of HIV-infected people in India, who are from the socioeconomically vulnerable group.

This is important as malnutrition has a major impact on the outcome of HIV disease as it not only increases mortality [12, 13] but also results in an impaired response to antiretroviral therapy [14]. Rajasekaran et al. showed that children who were severely malnourished at baseline, had a hazard ratio of 6.7 (0.9–49.4) for mortality after initiation of ART, compared to children who were normally nourished [14]. However nutritional recovery and growth after treatment of malnutrition is similar to that observed in HIV uninfected children, stressing the need for early recognition and management [15]. We explored this area in depth as none of the previous studies from India have examined the pattern and type of malnutrition in detail or attempted to study its correlation with age, gender, or immune status.

In our study, the prevalence of moderate to severe undernutrition was significantly different among the various age groups with higher rates of underweight among children less than 3 years of age and above 10 years of age. The increased risk of malnutrition in younger children may be due to a combination of factors like weaning from the breast, inadequate supplementary feeding, lose of passive immunity received from mother, and so forth, all leading to recurrent infections and a poorly nourished child. After the age of 10 years we see another rise in the rate of severe malnutrition and this could be explained by the fact that by this age many children start showing evidence of disease progression. In our study we observed that children above 10 years of age had lower mean CD4% and CD4 cell counts, indicating more advanced disease. The CD4 counts were lower in children with stunting and undernutrition compared to the age group as a whole—CD4% which are more stable than absolute counts also showed a decline. The proportion of children with “normal” nutrition decreased with advancing age. Wasting was relatively less prevalent in our cohort suggesting that malnutrition was of chronic onset and not an acute entity, unlike a report from Malawi where the commonest physical sign was wasting in more than 70% of the infected children [16].

While immune status and malnutrition showed a fair correlation, the presence of moderate stunting or undernutrition could not be used to predict disease severity very accurately. Among children with moderate to severe stunting, though the majority had low CD4%, almost one fourth of children in this group had CD4 >15%. Similarly, while underweight (WAZ < −2) children commonly had CD4 <15%, a quarter of these children also had a CD4% >15%. The sensitivity and specificity of predicting CD4% using either HAZ or WAZ was not very satisfactory. The area under the ROC curve for both WAZ and HAZ was in the range of 0.6–0.7, indicating poor diagnostic accuracy. Because malnutrition is common at all stages of HIV disease, stunting or undernutrition cannot be used as a surrogate marker for predicting disease stage or severity. Our study also highlights the fact that even at relatively early stages of the disease with higher CD4 counts, malnutrition is a substantial problem with over a third of children moderately or severely malnourished. By the time they reach a stage of advanced immunodeficiency, approximately three-quarters are stunted and underweight. Hence, there is a need for nutritional intervention at an early stage of the disease, as stunting may not be completely reversible if it is long standing.

The strengths of our study are that this was a group of well-characterized HIV-infected children representing all age groups. Both anthropometric and CD4 measurements were performed using standardized methods. There are a few limitations to our study. Since this study was cross-sectional in design, it was difficult to examine any temporal relationships between malnutrition and disease outcomes. As very sick children were not enrolled into this pediatric cohort study, there is the possibility of selection bias, thus under-estimating the actual prevalence of growth abnormalities. On the other hand, these children all came to a tertiary level centre to seek care and may therefore represent the more severely affected end of the spectrum. Further, the average age of this cohort was 6 years which is typical of HIV-infected children presenting to care in India. We have previously reported on infants with perinatally acquired HIV infection (virologically confirmed) who show rapid disease progression and die even before two years of age, mostly undiagnosed and uninitiated on treatment. Thus, we may have missed the most severely affected infants who never present to care till they are severely ill or moribund [17]. The findings from our study may not necessarily be reflective of the situation in other developing countries of Asia and Africa, where patterns of malnutrition vary. However, we have drawn attention to this important area which needs further research.

In summary, we have found that malnutrition (both stunting and underweight) is highly prevalent among HIV-infected children in India, at all ages and at all stages of HIV disease. Growth failure cannot be used as a surrogate marker to stage HIV disease as it occurs even at relatively higher CD4 levels. Malnutrition should be targeted early to ensure optimal response to ART and reduce early mortality. Future studies should also examine the impact of nutritional supplementation started at different stages of HIV disease on reducing HIV-related mortality and morbidity in children and in modifying long-term treatment outcomes.

## Figures and Tables

**Table 1:** Demographic profile of the study population.

Variable	Overall (*n* = 231) (mean ± SD)	Girls (*n* = 134) (mean ± SD)	Boys (*n* = 97) (mean ± SD)
Age (months)	71 ± 40	72 ± 39	69 ± 41.0
Weight (kgs)	14.4 ± 5.9	14.5 ± 5.7	14.4 ± 6.2
Height (cms)	99.7 ± 20.7	100.5 ± 18.8	98.7 ± 22.9
Body mass index (kg/m^2^)	14.2 ± 1.8	14.2 ± 1.8	14.2 ± 1.7
CD4 (%)	17.7 ± 10.2	18.5 ± 11.1	16.5 ± 8.6
CD4 Count (cells/mm^3^)	793 ± 614	785 ± 636	805 ± 583
Hemoglobin (gms/dl)	9.7 ± 2.1	9.8 ± 2.1	9.6 ± 2.0

**Table 2:** Gender wise prevalence of malnutrition among HIV-infected Children.

Anthropometric indices	Total number of Children *N* (%)	Girls *N* (%)	Boys *N* (%)	*p* value
Study population	231	134	97	
Underweight (WAZ < −2)	146 (63)	77 (58)	69 (71)	0.03
Stunting (HAZ < −2)	134 (58)	72 (54)	62 (64)	0.14
Wasting (WHZ < −2)	38 (16)	19 (14)	19 (20)	0.37

**Table 3:** Mean CD4% and CD4 cell count of children in different age groups as well as in those with underweight and stunting.

	<3 years	3–5 years	5–10 years	>10 years
*All children*				
CD4%	17.3 ± 6.6	19.2 ± 13.2	17.6 ± 9.4	15.8 ± 10.8
CD4 count (cells/mm^3^)	1120 ± 619	1036 ± 778	666 ± 472	423 ± 345
*Underweight*				
CD4%	16.9 ± 7.0	15.8 ± 7.9	14.6 ± 8.6	11.4 ± 7.8
CD4 count (cells/mm^3^)	1117 ± 638	842 ± 591	581 ± 483	305 ± 276
*Stunted*				
CD4%	17.0 ± 6.9	16.2 ± 8.2	15.5 ± 9.2	14.0 ± 10.1
CD4 count (cells/mm^3^)	1180 ± 643	888 ± 577	644 ± 537	337 ± 277

**Table 4:** Prevalence of underweight, stunting, and wasting at different levels of immunodeficiency.

Malnutrition scores	Levels of immunodeficiency		
	CD4 < 15%	CD4 15–25%	CD4 > 25%
	*N* = 79	*N* = 82	*N* = 33
	*n* (%)	*n* (%)	*n* (%)
WAZ < −2	60 (76)*	50 (61)	11 (33)
HAZ < −2	56 (71)*	44 (54)	14 (42)
WHZ < −2	12 (15)	15 (18)	3 (9)

*P < .001 across levels of immunodeficiency.
